# Prospective evaluation of 92 protein biomarkers for early detection of endometrial cancer

**DOI:** 10.1002/ijc.35428

**Published:** 2025-04-03

**Authors:** Victoria Cooley, Renée Turzanski Fortner, Trasias Mukama, Sabine Naudin, Valeria Pala, Laure Dossus, Inger T. Gram, Karina Standahl Olsen, Maria‐Jose Sánchez, Pernilla Israelsson, Naomi Allen, Hilde Langseth, Rudolf Kaaks

**Affiliations:** ^1^ Division of Cancer Epidemiology German Cancer Research Center (DKFZ) Heidelberg Germany; ^2^ Medical Faculty Heidelberg Heidelberg University Heidelberg Germany; ^3^ Department of Research Cancer Registry of Norway, Norwegian Institute of Public Health Oslo Norway; ^4^ Makerere University School of Public Health Kampala Uganda; ^5^ Paris‐Saclay University, UVSQ, Inserm, Gustave Roussy, CESP Villejuif France; ^6^ Epidemiology and Prevention Unit Fondazione IRCCS Istituto Nazionale dei Tumori di Milano Milan Italy; ^7^ Nutrition and Metabolism Branch International Agency for Research on Cancer, World Health Organization Lyon France; ^8^ Faculty of Health Sciences, Department of Community Medicine UiT The Arctic University of Norway Tromsø Norway; ^9^ Escuela Andaluza de Salud Pública (EASP) Granada Spain; ^10^ Instituto de Investigación Biosanitaria ibs.GRANADA Granada Spain; ^11^ Centro de Investigación Biomédica en Red de Epidemiología y Salud Pública (CIBERESP) Madrid Spain; ^12^ Department of Radiation Sciences, Oncology Umeå University Umeå Sweden; ^13^ Nuffield Department of Population Health University of Oxford Oxford UK; ^14^ Department of Epidemiology and Biostatistic s School of Public Health, Imperial College London London UK

**Keywords:** biomarkers, early detection, endometrial cancer

## Abstract

The human epididymis protein 4 (HE4) remains the best available endometrial cancer (EC) biomarker; however, its discrimination between cases and cancer‐free individuals is limited and might be improved when combined with other protein markers. We evaluated the discrimination capacity of 92 proteins as potential early detection biomarkers for EC in nested case–control studies in the European Prospective Investigation into Cancer and Nutrition (EPIC) (63 cases, 123 controls) and Janus (75 cases, 146 controls) cohorts, evaluating blood samples taken ≤2 years prior to diagnosis. Proteins were measured with the Olink Target 96 Oncology II panel assays. Areas under the receiver operating characteristic curves (AUCs) were calculated using logistic regression. The discrimination between cases and controls of top‐performing proteins was modest (EPIC: HE4, CA125, CAIX, and S100A4; Janus: HE4, CA125, FURIN, CXCL13, and IL6; AUC range: 0.65 [S100A4], 0.76 [HE4, EPIC] within 0 to <12 months of blood collection) and decreased as the time between blood draw and cancer diagnosis increased (12–24 months AUC range: 0.49 [S100A4], 0.69 [CA125, Janus]). The combination of these other markers with HE4 did not improve discrimination. HE4 and other candidate proteins had limited discrimination between EC cases and controls and hence do not appear to be useful for early detection of this disease in women at average population risk.

## Graphical abstract

What's new?

In this prospective study, the authors evaluated the discrimination capacity of 92 cancer‐related proteins as potential early detection biomarkers for endometrial cancer using blood samples from two large‐scale European population cohorts (EPIC and Janus). The human epididymis protein 4 (HE4) and the antigen CA125 demonstrated the best—though limited—discrimination capacity between cases and controls across both cohorts up to two years prior to diagnosis. Discrimination waned as the time from blood draw to diagnosis increased, and the combination of other markers with HE4, currently the best endometrial cancer biomarker available, did not result in improvements in discriminative performance.

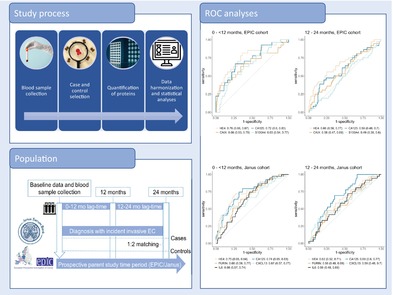

**Abbreviations**
AEH: atypical endometrial hyperplasia
aROCs: covariate‐adjusted receiver operating curves
AUC: area under the curve
BMI: body mass index
CIs: confidence intervals
CRN: Cancer Registry of Norway
EC: endometrial cancer
EIN: endometrial intraepithelial neoplasia
EPIC: European Prospective Investigation into Cancer and Nutrition
HE4: human epididymis protein 4
PEA: proximity extension assay
SE95: sensitivities at 95% specificity
TVUS: transvaginal ultrasound

## 1 INTRODUCTION

Endometrial cancer (EC) is the most common gynecological cancer in many high‐income countries. The earlier detection of EC could improve survival outcomes, but currently, there is no population‐based screening method recommended for this cancer. Current screening and targeted prevention strategies are restricted to high‐risk women (i.e., Lynch syndrome) and include annual transvaginal ultrasound (TVUS) for screening and radical hysterectomy for prevention.^1^ While the early recognition of symptoms, such as abnormal vaginal bleeding or discharge, often leads to diagnosis at an earlier stage (localized: 69% of cases; regional: 17% of cases) where the 5‐year relative survival is favorable (localized: 95%; regional: 70%), the 5‐year relative survival estimates for those with advanced disease are only 18%.^2^, ^3^ In Norway, the 5‐year relative survival for localized disease is 98.7%, 68.0% for regional, and 30.1% for distant disease.^4^

Postmenopausal bleeding, which occurs in the vast majority of women with EC, lacks sufficient specificity, and only 9% of women with bleeding have a resulting EC diagnosis following clinical investigation.^5^ Endometrial biopsies, which are often used in combination with TVUS as a diagnostic measure, are associated with varying degrees of pain and discomfort and have a failure rate of approximately 11%.^6^ The specificity of TVUS imaging is also lower, at 86% for an endometrial thickness cutoff of 3.0–5.9 mm.^7^ The use of diagnostic tests that rely upon results collected from blood, cervicovaginal fluid, and urine has the potential to provide a minimally invasive approach to the earlier detection of EC and also to reduce the number of unnecessary endometrial biopsies.^8^ Such methods, however, are not available for clinical use, and additional prospective studies are needed to generate evidence to support their utility in clinical practice.^8^ The justification of mainstream screening practices is often not deemed worthy from a mortality reduction perspective when considering the possibility of producing unnecessary concern over many false‐positive tests and the associated resourcing and cost implications.

The human epididymis protein 4 (HE4) is the best EC biomarker found so far in clinical studies,^9^, ^10^, ^11^ although other blood‐based biomarkers such as antigens CA125, CA72‐4, and CA19‐9,^12^ inflammation markers,^13^ angiogenesis factors,^14^ and other proteins^1^, ^15^ have been identified as being associated with EC. HE4 concentrations, however, have also been shown to be elevated in association with other cancers (i.e., pancreatic and ovarian)^11^ and this biomarker has been shown to provide only limited discrimination between EC patients and non‐cases (area under the curve [AUC] range: 0.68–0.77).^16^

One way to potentially improve the specificity and overall discrimination capacity for the detection of EC is to use multi‐protein biomarker panels that include HE4 plus other proteins. This motivates the search for the identification and/or validation of further candidate detection markers, using modern proteomics and multi‐protein assay platforms. Two previous retrospective case–control studies utilized different multiplex assays of cancer‐related markers based on the proximity extension assay (PEA) technology from Olink Proteomics (Target Oncology panels) and identified proteins (<10 proteins per study from final analyses; overlapping protein: MMP‐7) associated with EC.^17^, ^18^ Furthermore, using the same panel, a study nested within the prospective European Prospective Investigation into Cancer and Nutrition (EPIC) cohort assessed and partially confirmed the potential of several of this panel's markers for the early detection of ovarian cancer, up to 9 months before diagnosis.^19^

A limitation of all of the studies on the identification or validation of early detection biomarkers for EC is that they have compared protein profiles between prevalent, clinically manifest EC samples and those without cancer. It thus remains unclear whether, or to what extent, protein markers may be used for detecting EC before the appearance of clinical symptoms. In considering the low relative 5‐year survival rates for women diagnosed with advanced disease, there is a need to identify additional biomarkers that can aid in identifying this disease before the manifestation of major symptoms, in order to offer curative treatment. We here present findings from a first prospective evaluation of proteins as potential early detection biomarkers for EC, using nested case–control studies in two large‐scale population cohorts. We used the Olink Target 96 Oncology II panel and assessed the discriminative capacity of a total of 92 cancer‐related proteins, including HE4 and CA125, for the prospective detection of EC through measurements in plasma and serum samples that had been collected up to 2 years prior to diagnosis.

## 2 METHODS

### 2.1 Case–control studies nested within the EPIC and Janus cohorts

The sample for the current study includes nested case–control studies within the EPIC and the Norwegian Janus Serum Bank (Janus) cohorts. EPIC is an ongoing multi‐center prospective cohort study that aims to investigate the relationship between nutrition and cancer.^20^ The enrollment of 519,978 participants (226,673 women who provided a blood sample) took place between 1992 and 2000 across 23 centers in 10 countries. Data were collected via questionnaire and anthropometric measures, and blood samples were collected. Janus is a large prospective population‐based biobank that was established in 1973 and includes blood samples from 318,628 Norwegians (152,491 women).^21^, ^22^ Approximately 90% of the cohort participated in Norwegian Regional Health Studies (years 1972–2003) and has questionnaire‐based data on lifestyle, as well as anthropometric, and biochemical data available; and the remainder consists of Red Cross blood donors.

### 2.2 Sample selection and data collection

Cases were defined as women diagnosed with incident invasive EC (ICD‐O code: C541) within 24 months of blood collection. Women were excluded if they had a previous cancer diagnosis (with the exception of non‐melanoma skin cancer) prior to blood draw. Incidence density sampling with a 1:2 case–control matching ratio was utilized. Tumors were classified as Type I or Type II in line with the classifications previously implemented.^23^

#### 2.2.1 European Prospective Investigation into Cancer and Nutrition

Within the EPIC cohort, cases were identified through record linkages with regional cancer and pathology registries (all countries except France, Germany, and Naples, Italy) and active follow‐up and verification of self‐reports (France, Germany and Naples, Italy). Women were excluded if they had reported a hysterectomy at the time of blood draw. Cases and controls were matched on the following characteristics at the time of blood collection: study center, menopausal status (premenopausal, postmenopausal, unknown), exogenous hormone use, age at blood collection (±6 months), time of the day of blood collection (±1 h), time between blood draw and last consumption of foods or drinks (<3, 3–6, >6 h, unknown), and for premenopausal women, phase of menstrual cycle. Tumor characteristics such as stage (available as localized, metastatic regional, and metastatic categories), histology, and grade were obtained from pathology reports and cancer registries. Information on additional epidemiologic risk factors such as smoking status, body mass index (BMI), menopausal status, and oral contraceptive use was obtained from baseline questionnaires and measurements. We excluded women based on the following characteristics in sequential order: 21,205 with prevalent cancers, 35,970 with hysterectomy, 53 with incomplete data with no lifestyle or dietary information available, and 2385 with no follow‐up data available. The final sample from EPIC included 63 cases and 123 matched controls.

#### 2.2.2 Janus

Cases were identified through linkage with the Cancer Registry of Norway (CRN), which has had mandatory reporting of cancer cases since 1953.^24^ Cases and controls were matched on the following characteristics at the time of blood collection: source of blood collection (health examination, blood donors), age (within ±1 year from age of case at blood draw), date of blood draw (within ±3 months from date of blood draw of case), and county of residence. Information on tumor characteristics such as stage (available as localized, regional metastasis, and distant metastasis categories), histology, and grade was obtained from the CRN. Information on additional epidemiologic risk factors such as smoking status and BMI was obtained from the Norwegian Regional Health Studies.^21^ A total of 2105 women were excluded due to prevalent cancers (follow‐up data available for all Janus members via registries). The final sample included 75 cases and 146 matched controls.

### 2.3 Laboratory assays

The Olink Target 96 Oncology II panel assays were performed in an Olink® certified laboratory at the German Center for Environmental Health (Helmholtz Zentrum München), Neuherberg, Germany. Samples were analyzed in batches, with samples from matched case–control sets included in the same batch in a randomized and blinded order. Results were reported in “normalized protein expression” values on the log2 scale. For small proportions of study participants, and for relatively few proteins, PEA measurements fell below the detection limit (EPIC: FADD [18.1%], VIM [3.0%], CEACAM5 [3.5%], CA125 [3.5%]; Janus: TXLNA: [2.5%], FADD [0.4%], VIM [0.4%], CEACAM5 [0.8%], CA125 [1.2%], FCRLB [5.8%]). When assay results were below the limit of detection, we assigned values equal to the midpoint between zero and the lower limit of detection.

### 2.4 Statistical analysis

To assess the discrimination performance of the proteins, covariate‐adjusted receiver operating curves (aROCs) were generated for protein‐related risk scores developed from unconditional logistic regression models,^25^, ^26^, ^27^ and the AUC with 95% confidence intervals (CIs) was calculated for each protein, separately by cohort and lag‐time interval (0 to <12 and 12 to 24 months). All aROCs were adjusted for age at blood draw, and EPIC aROCs were additionally adjusted for menopausal status. Proteins that had an AUC of ≥0.65 for EC cases in the 0 to <12 months lag‐time interval compared to controls were selected for further evaluation for sensitivities at 95% specificity (SE95). Spearman's partial correlations, adjusting for age at blood draw and cohort, were calculated within case and control groups to assess relationships between the selected proteins (Supporting Information Figure F1). Locally Estimated Scatterplot Smoothing was used to produce fitted lines in the visualization of the top performing protein levels by time before EC diagnosis (Supporting Information Figure F3). Multi‐marker models, in which the selected proteins were added singly to a model containing HE4, were developed to assess any improvement in discriminative performance. Likelihood ratio tests were utilized to assess any improvements in model fit. Optimism‐corrected AUCs were calculated through 1000 bootstrap iterations, to account for overfitting in the multi‐marker models. Statistical significance was evaluated at the 0.05 alpha level. All analyses were executed using R (4.3.0) for Windows.^28^

## 3 RESULTS

The characteristics of cases and controls in the EPIC and Janus cohorts are presented in Table 1. The median age at blood collection was 58 years for cases and controls (interquartile range IQR: 54, 63) in the EPIC cohort and 50 for cases (IQR: 43, 54) and 49 for controls (IQR: 43, 54) in the Janus cohort (Table 1). The median age at EC diagnosis was 59 (IQR: 55, 63) in EPIC and 51 (IQR: 43, 55) in Janus. In both cohorts, the majority of cases were diagnosed at the localized stage (EPIC: 75%; Janus 79%).

**TABLE 1:** Characteristics of cases and controls in the EPIC and Janus cohorts.

Characteristic	EPIC		Janus	
	Case, *N* = 63^a^	Control, *N* = 123^a^	Case, *N* = 75^a^	Control, *N* = 146^a^
Age at blood draw	58 (54, 63)	58 (54, 63)	50 (43, 54)	49 (43, 54)
BMI	26.8 (23.3, 30.3)	25.0 (23.0, 27.8)	25.3 (23.5, 30.1)	24.5 (22.4, 27.0)
Unknown			14	24
Smoking status				
Current	8 (13%)	18 (15%)	24 (39%)	41 (34%)
Former	14 (23%)	19 (16%)	8 (13%)	20 (16%)
Never	39 (64%)	82 (69%)	29 (48%)	61 (50%)
Unknown	2	4	14	24
Age at diagnosis	59 (55, 63)		51 (43, 55)	
Histology				
Type I	55 (87%)		64 (85%)	
Type II	8 (13%)		2 (2.7%)	
Sarcomas	0 (0%)		9 (12%)	
Grade				
Well differentiated	17 (35%)		5 (63%)	
Moderately differentiated	19 (40%)		2 (25%)	
Poorly differentiated/undifferentiated	8 (17%)		1 (13%)	
Not determined	4 (8.3%)		0 (0%)	
Unknown	15		67	
Stage				
Localized	30 (75%)		58 (79%)	
Regional/metastatic	10 (25%)		15 (21%)	
Unknown	23		2	
Lag‐time category (months)				
0 to <12	27 (43%)		39 (52%)	
12 to 24	36 (57%)		36 (48%)	
Lag‐time (months)	13 (8, 19)		12 (7, 19)	

Abbreviations: BMI, body mass index; EPIC, European Prospective Investigation into Cancer and Nutrition.

^a^ Median (IQR), *n* (%).

The discrimination of HE4 and CA125 was similar between cohorts, with slightly higher AUC values across both lag‐time intervals for HE4 in the EPIC cohort (EPIC: 0 to <12 months: 0.76 [0.66, 0.87] and 12 to 24 months: 0.66 [0.56, 0.77] compared with the Janus cohort: 0 to <12 months: 0.75 [0.65, 0.84] and 12 to 24 months: 0.62 [0.52, 0.71]). AUC values for CA125 were slightly higher in the Janus cohort across both lag‐time intervals (Janus: 0 to <12 months: 0.74 [0.65, 0.83] and 12 to 24 months: 0.69 [0.6, 0.77]; EPIC: 0 to <12 months: 0.72 [0.6, 0.83] and 12 to 24 months: 0.59 [0.48, 0.7]). Overall, HE4 generally had the highest discrimination within both cohorts and across lag‐times, with the exception of the 12–24 months lag‐time in the Janus cohort where CA125 had a slightly higher AUC (Table 2; AUC [95% CI] values for all individual proteins found in Supporting Information Table T1).

**TABLE 2:** Multi‐marker models featuring the addition of top‐performing proteins^a^ to a model with HE4 in the EPIC and Janus cohorts.

Model	EPIC				Janus			
	0 to <12, AUC (95% CI)	Optimism corrected AUC	12 to 24, AUC (95% CI)	Optimism corrected AUC	0 to <12, AUC (95% CI)	Optimism corrected AUC	12 to 24, AUC (95% CI)	Optimism corrected AUC
HE4	0.76 (0.66, 0.87)	NA	0.66 (0.56, 0.77)	NA	0.75 (0.65, 0.84)	NA	0.62 (0.52, 0.71)	NA
CA125	0.72 (0.6, 0.83)	NA	0.59 (0.48, 0.7)	NA	0.74 (0.65, 0.83)	NA	0.69 (0.6, 0.77)	NA
CAIX	0.66 (0.53, 0.79)	NA	0.58 (0.47, 0.69)	NA	—	—	—	—
S100A4	0.65 (0.54, 0.77)	NA	0.49 (0.38, 0.6)	NA	—	—	—	—
FURIN	—	—	—	—	0.68 (0.59, 0.77)	NA	0.58 (0.48, 0.69)	NA
CXCL13	—	—	—	—	0.67 (0.57, 0.77)	NA	0.59 (0.48, 0.7)	NA
IL6	—	—	—	—	0.66 (0.57, 0.74)	NA	0.59 (0.48, 0.69)	NA
Marker combinations								
HE4 + CA125	0.79 (0.68, 0.9)	0.76	0.67 (0.56, 0.77)	0.62	0.78 (0.69, 0.86)	0.76	0.7 (0.61, 0.78)	0.66
HE4 + CAIX	0.8 (0.7, 0.9)	0.77	0.69 (0.59, 0.8)	0.65	—	—	—	—
HE4 + S100A4	0.82 (0.74, 0.9)	0.79	0.67 (0.56, 0.78)	0.62	—	—	—	—
HE4 + FURIN	—	—	—	—	0.76 (0.67, 0.84)	0.74	0.63 (0.53, 0.73)	0.58
HE4 + CXCL13	—	—	—	—	0.75 (0.65, 0.84)	0.73	0.62 (0.52, 0.73)	0.58
HE4 + IL6	—	—	—	—	0.75 (0.66, 0.84)	0.73	0.62 (0.52, 0.72)	0.57

*Note*: All models are additionally adjusted for age at blood draw and additionally menopausal status at blood draw for the EPIC cohort. Optimism‐corrected AUC values are presented to account for overfitting.

^a^ Only proteins with AUCs ≥0.65 are presented.

**FIGURE 1:**
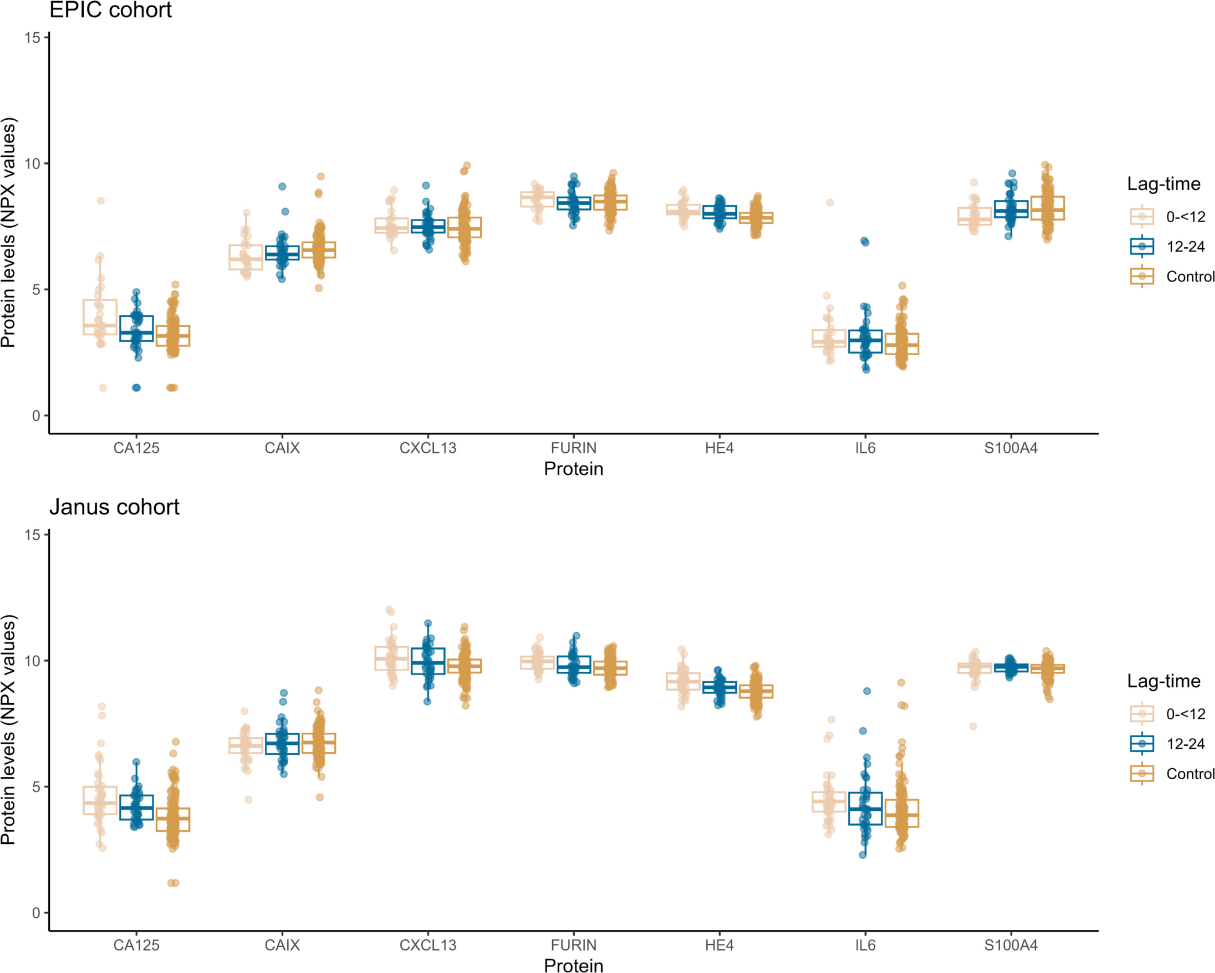
Distributions of proteins in the EPIC and Janus cohorts, stratified by lag‐time category. The proteins above displayed an AUC value of ≥0.65 for cases in the 0 to <12 months lag‐time interval and controls, and were selected for further analysis. AUC, area under the curve; EPIC, European Prospective Investigation into Cancer and Nutrition.

**FIGURE 2:**
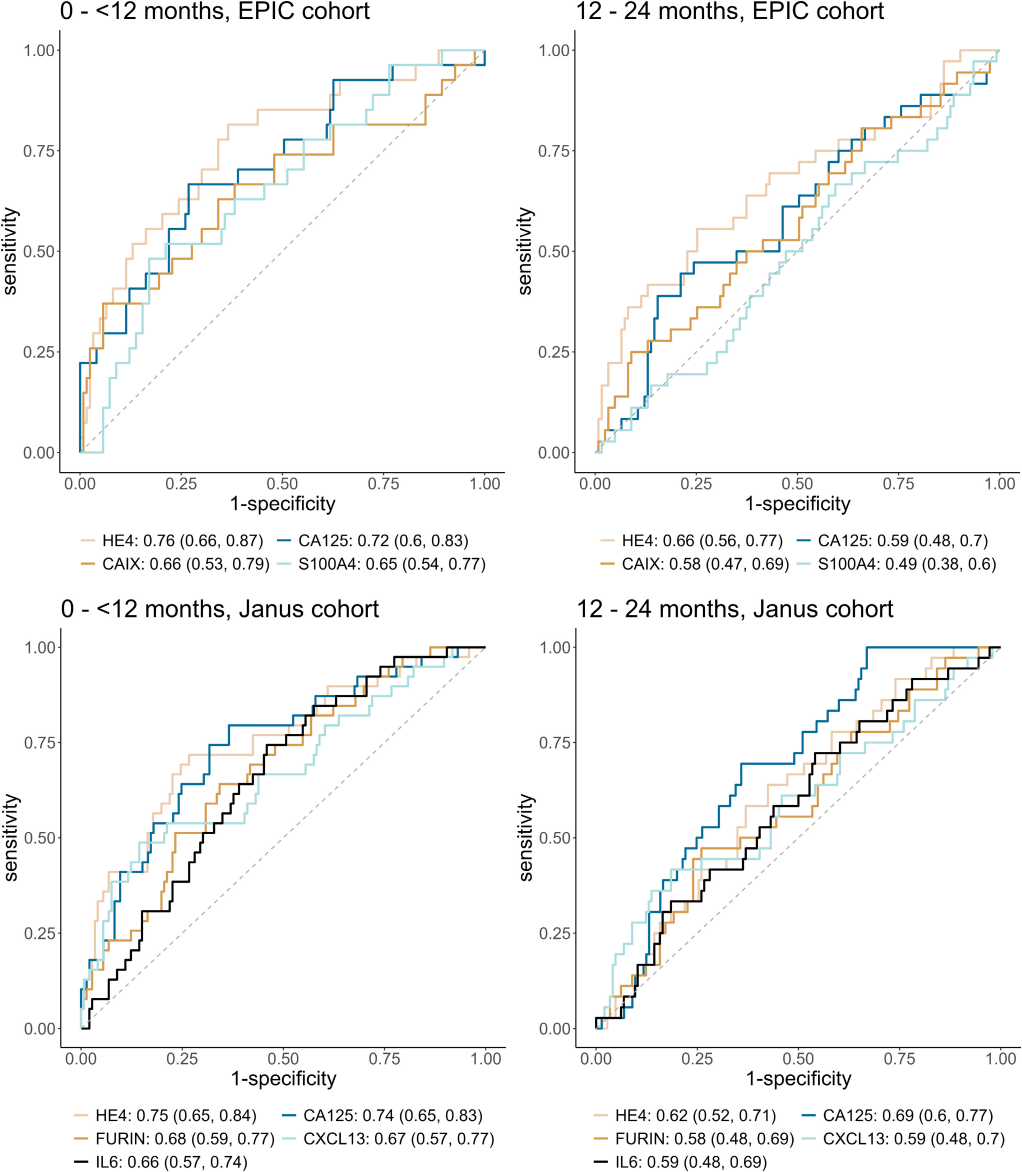
Cohort‐specific ROC curves depicting the discriminative performance (AUC [95% CI]) of proteins with AUCs ≥0.65 for cases in the 0 to <12 months lag‐time interval and controls. ROC curves for these top performing proteins in the 12–24 lag‐time interval are also displayed. For both the EPIC and Janus cohorts, AUCs are adjusted for age at blood draw, and additionally menopausal status for the EPIC cohort only. AUC, area under the curve; EPIC, European Prospective Investigation into Cancer and Nutrition; ROC, receiver operating characteristics.

In addition to HE4 and CA125, two other proteins in the EPIC cohort (CAIX and S100A4) and three other proteins in the Janus cohort (FURIN, CXCL13, and IL6) had an AUC of ≥0.65 for the discrimination between cases and controls in the 0 to <12 months lag‐time interval; the distributions of the proteins by lag‐time are shown in Figure 1. For both cohorts, the discrimination of these proteins substantially decreased as the time between blood draw and cancer diagnosis increased (i.e., HE4 in EPIC cohort: 0 to <12 months: 0.76 [0.66, 0.87] and 12 to 24 months: 0.66 [0.56, 0.77]; FURIN in the Janus cohort: 0 to <12 months: 0.68 [0.59, 0.77] and 12 to 24 months 0.58 [0.48, 0.69]) (Figure 2; further ROC curves presented in Supporting Information Figure F2). The SE95 values for these proteins also displayed a similar decline by lag‐time within both cohorts, and sensitivities approached zero for the 12–24 months category (Table 3).

**TABLE 3:** Sensitivities at 95% specificity for top performing proteins by lag‐time (selection criteria is mentioned in the methods section) in the EPIC and Janus cohorts.

Protein	Sensitivity at 95% specificity by lag‐time (months)			
	EPIC, 0 to <12	EPIC, 12 to 24	Janus, 0 to <12	Janus, 12 to 24
HE4	0.33 (0.11, 0.52)	0.22 (0.08, 0.47)	0.33 (0.08, 0.51)	0.08 (0, 0.19)
CA125	0.26 (0.11, 0.44)	0.06 (0, 0.17)	0.18 (0.08, 0.41)	0.03 (0, 0.14)
CAIX	0.26 (0.11, 0.52)	0.14 (0, 0.31)	0.15 (0.05, 0.28)	0.06 (0, 0.19)
S100A4	0 (0, 0.3)	0.06 (0, 0.14)	0.08 (0, 0.21)	0.06 (0, 0.17)
FURIN	0.04 (0, 0.19)	0.03 (0, 0.11)	0.15 (0.05, 0.33)	0.08 (0, 0.19)
CXCL13	0.15 (0, 0.3)	0.03 (0, 0.14)	0.18 (0.08, 0.46)	0.19 (0, 0.33)
IL6	0.07 (0, 0.3)	0.11 (0.03, 0.22)	0.08 (0, 0.21)	0.03 (0, 0.14)

*Note*: Non‐overlapping proteins are included for comparability purposes.

Multi‐marker models featuring the addition of the top performing proteins (listed above) to a model with HE4 were developed (Table 2). In the 0 to <12 lag‐time interval in the EPIC cohort, the separate addition of CAIX, S100A4, and CA125 to a model with HE4 alone resulted in statistically significant improvements in model fit, but only minor increases in AUC values (Table 2). A similar finding was observed in the Janus cohort for the addition of CA125 in both lag‐times (i.e., Janus 0 to <12 months: HE4 AUC = 0.75, HE4 + CA125 overoptimism corrected AUC: 0.76; Table 2).

## 4 DISCUSSION

In this first prospective study to evaluate 92 proteins on the Olink Oncology II panel as early detection biomarkers for EC, HE4 and CA125 demonstrated the best discrimination between cases and controls in the EPIC and Janus cohorts for cancers diagnosed within a year and between 1 and 2 years of a diagnosis. They were the only two proteins that consistently showed discrimination capacity (i.e., lower 95% confidence limits >0.50) in both cohorts, despite differences in the distributions of the age at diagnosis. These findings in the pre‐diagnostic setting confirm those of previous, cross‐sectional case–control comparisons based on prevalent cases and control women, where both HE4^9^, ^10^, ^11^ and CA125^12^, ^29^ were also found to be the two best discriminating markers. For both HE4 and CA125, but also the other proteins with AUCs ≥0.65 in at least one of the cohorts for blood draws 0 to <12 months prior to diagnosis (CAIX, S100A4, FURIN, CXCL13, and IL6), discrimination waned with longer time between blood draw and diagnosis (i.e., 12–24 months). Finally, upon the addition of these proteins to a model with HE4, only minimal increases in discrimination were observed across both cohorts and lag‐times, and again their joint discrimination capacity decreased for the 12–24 months lag‐time.

Of the seven proteins suggesting discrimination potential in our study, only HE4 and IL6 were identified previously as being associated with EC in one^17^ of two cross‐sectional studies in Sweden or Italy, also using the Olink technology.^17^, ^18^ Other retrospective and nested case–control studies have utilized enzyme‐linked and bead‐based immunoassays for the serum and plasma quantification of IL6^13^, ^30^ and spectrophotometry‐based methods for the uterine lavage measurement of pro‐protein convertases, notably FURIN.^31^ IL6 is a pro‐inflammatory cytokine that is released by macrophages within adipose tissue and has been found to be involved in tumorigenesis through multiple pathways.^32^ In these studies, IL6 exhibited potential in differentiating EC cases from controls, but associations became attenuated and did not persist after the adjustment for BMI.^13^, ^30^ Most of the previous studies included plasma samples from prevalent EC cases and cancer‐free control women, and the discrimination potential for proteins, as observed in these studies, might not necessarily translate well to pre‐diagnosis settings, to identify cancer before the manifestation of any other symptoms.

While other proteins such as Mk^17^ and COL9A1^18^ were identified as being associated with EC on the Target 96 Oncology II^17^ or III^18^ panels, the study from Sweden utilized four other Olink Multiplex assays (CVD II, CVD III, INF I, NEU I; proteins PRSS8, ADM, MMP‐7, ST2, VEGF‐A, and HGF identified)^17^ and the study from Italy utilized the Target 96 Immuno‐Oncology panel (proteins Gal‐1, Gal‐9, MMP7, and FASLG identified).^18^ The Target 96 Oncology II panel (used in our present study) and the Oncology III panel (used in the study from Italy) feature entirely different proteins, which also makes any direct comparisons of study findings impossible. Overall, the endometrial cases in our study were younger than those of the two earlier studies mentioned above. In addition, our study included a mix of endometrioid and other tumor types, while the study from Italy included endometrioid adenocarcinomas.^18^

When interpreting the results of this study, it is important to consider the temporal trajectory of the diagnosis of EC from the development of initial symptoms and precursor lesions. EC often develops from endometrial intraepithelial neoplasia (EIN) or atypical endometrial hyperplasia (AEH). During the development of AEH, the PTEN gene undergoes somatic mutations and then mutations in the ARID1A gene and inactivation of TGF‐β are involved in the ultimate progression into invasive endometrioid carcinoma.^33^, ^34^, ^35^ A hysterectomy is typically the primary treatment unless a fertility‐sparing pharmacologic treatment in the form of pro‐gestational agents is desired.^36^ Approximately 30%–50% of women with EIN‐AEH who have a hysterectomy are found to have EC.^36^ Endometrial sampling is used for women who desire to undergo fertility‐sparing treatment to definitively rule out EC and a histologic assessment within 3–6 months of therapy is given.^36^ The timeline that spans the transition from hyperplasia to AEH to EC is unclear. The relative timing of each interval is not fully defined, with the potential for reversion of the hyperplasia with the usage of progestin therapy. In considering the complexity of the trajectories mentioned above, and the additional potential to dismiss intermenstrual and post‐menopausal bleeding as a benign condition and delay diagnosis,^5^, ^37^ additional investigation is needed to better understand the time interval between early symptom development and diagnosis. Such an improved understanding can inform future early detection work, helping to identify an optimal window of time following symptom presentation where a diagnosis is plausible and allowing the investigation of other marker pathways.

We observed a substantial decrease in discrimination between subsequent cases and controls, comparing those providing a blood sample proximate to diagnosis (i.e., 0–12 months) to those providing a sample more distant from diagnosis (i.e., 12–24 months). The lack of prospective studies in the current literature and the complexity around the length of the interval surrounding the experience of symptoms and development of precursor lesions to EC make it challenging to describe the relationship between the decline in discrimination and the biological changes occurring in the pre‐diagnostic phase and progression of the development of neoplasm. One study featuring a follow‐up period of 20 years of women with endometrial hyperplasia reported a 20‐year progression to endometrial carcinoma risk of 28% among those with AEH.^38^ Measurements fell below the detection limit for relatively few proteins and subjects in our study, and we would expect that this decline in discrimination over time would not be largely reflective of limitations in the protein assay used. The evaluation of future assays with lower limits of detection, where lower but meaningful levels of protein could be detected among cases, would be needed to fully elucidate this point.

In previous work assessing the potential of early detection biomarkers for ovarian cancer, such a decrease in discrimination with increasing time between blood draw and diagnosis has also been observed.^19^, ^39^ In a previous nested case–control study conducted within the EPIC cohort using the same protein panel, HE4 and CA125 were found to have the best discrimination in the 0 to ≤9 months lag‐time interval (AUC [95% CI]: 0.86 [0.82, 0.89] and 0.84 [0.81, 0.87], respectively) but performance substantially decreased for the >9–18 months lag‐time.^19^ The detection of HE4 and CA125 as the best performing early detection biomarkers for both endometrial and ovarian cancer is expected, given the presence of the endometrioid histotype in ovarian cancer and that both cancers are gynecological in nature. Proteins such as FOLR1, KLK11, MDK, WISP1, CXCL13, MSLN, and ADAM8 were identified in this prior study, and none overlapped with the current study (with the exception of CXCL13 in Janus). Further studies featuring a larger number of cases are needed to confirm the specific utility of these proteins for ovarian cancer earlier detection.^19^

This study has a few limitations. There is a potential for between‐person and between‐study heterogeneity in diagnosis dates. Also, no data were available regarding the development and experience of symptoms and their timing relative to blood draw in either of the two cohorts. Future work incorporating symptom data into multi‐marker discrimination models featuring HE4, CA125, and other promising proteins could be of potential benefit for the earlier detection of EC. This study was limited to cases diagnosed within the 2 years following blood collection, and the assessment of lag‐time intervals longer than 2 years for additional marker pathways demonstrating potential would be of interest for future studies. The limited sample size and small number of cases in this study made it difficult to explore more granular lag‐time categories and develop more complex multi‐marker models, and further useful discriminatory proteins might have also been missed. Given that the discrimination potential of many proteins was assessed, the likelihood of a Type 1 error is increased, though our identification of HE4 and CA125 in both cohorts is consistent with previous literature.

HE4 and CA125 only found in both of the EPIC and Janus cohorts displayed the highest, though limited, discrimination as EC early detection biomarkers across both of the lag‐times assessed. Overall, discrimination decreased with increasing lag‐time, and the combination of single markers with HE4 did not result in any improvement in discriminative performance. Additional studies including larger sample sizes, longer lag‐time intervals, and most importantly the investigation of alternative marker pathways, together with risk factor, symptom, and imaging data where available, are needed to identify biomarkers to improve the EC diagnosis lead time.

## AUTHOR CONTRIBUTIONS

**Victoria Cooley:** Writing – original draft; visualization; formal analysis; data curation; software. **Renée Turzanski Fortner:** Conceptualization; writing – review and editing; supervision; resources; investigation; methodology. **Trasias Mukama:** Writing – review and editing. **Sabine Naudin:** Writing – review and editing; resources. **Valeria Pala:** Writing – review and editing; resources. **Laure Dossus:** Writing – review and editing; resources. **Inger T. Gram:** Writing – review and editing; resources. **Karina Standahl Olsen:** Writing – review and editing; resources. **Maria‐Jose Sánchez:** Writing – review and editing; resources. **Pernilla Israelsson:** Writing – review and editing; resources. **Naomi Allen:** Writing – review and editing; resources. **Hilde Langseth:** Writing – review and editing; resources. **Rudolf Kaaks:** Conceptualization; funding acquisition; writing – review and editing; resources; supervision; investigation; methodology; validation; project administration.

## FUNDING INFORMATION

The article is supported by Barrie Dalgleish Centre for Myeloma and Related Blood Cancers and Leukemia and Lymphoma Society.

## CONFLICT OF INTEREST STATEMENT

The authors declare no conflicts of interest.

## ETHICS STATEMENT

All EPIC study participants have given written consent for future analyses of their blood samples for research purposes. This study was approved by the International Agency for Research on Cancer (IARC, Lyon, France) Ethics Committee. The use of Janus in this study was approved by the Norwegian Regional Committee for Medical and Health Research Ethics (REC no. 509876). The Janus donors have given a broad consent for the use of the samples in cancer research.

## Supporting information


**Data S1.** Supporting Information.

## Data Availability

The data that support the findings of this study are available from the corresponding author upon reasonable request.
