# Supplementary material for: Prospective evaluation of 92 protein biomarkers for early detection of endometrial cancer
Source: Int J Cancer. 2025 Apr 3;157(3):480–9. doi: 10.1002/ijc.35428 (PMC12141982; doi:10.1002/ijc.35428)

**Prospective evaluation of 92 protein biomarkers for early detection of endometrial cancer**

Victoria Cooley, Renée Turzanski Fortner, Trasias Mukama, Sabine Naudin, Valeria Pala, Laure Dossus, Inger T. Gram, Karina Standahl Olsen, Maria-Jose Sánchez, Pernilla Israelsson, Naomi Allen, Hilde Langseth, Rudolf Kaaks

## Table of contents

Supplemental Figure F1. Spearman's rank partial correlations for all pairwise marker combinations ................................. 2

Supplemental Figure F2. ROC curves depicting the discriminative performance (AUC (95% CI)) of top performing proteins that did not overlap between the EPIC and Janus cohorts (i.e, top performing proteins found in Janus, but not in EPIC and vice versa)........................................................................................................................................ 4

**Supplemental Table T1.** Discriminative performance (AUC (95% CI)) of all proteins across both the EPIC and Janus cohorts and both lag-times .......................................................................................................................... 5

Supplemental Figure F3. Top performing protein levels by time before endometrial cancer diagnosis. .............................. 11

**Supplemental Figure F1.** Spearman's rank partial correlations for all pairwise marker combinations, adjusting for age at blood draw and cohort⁺

*Cases*

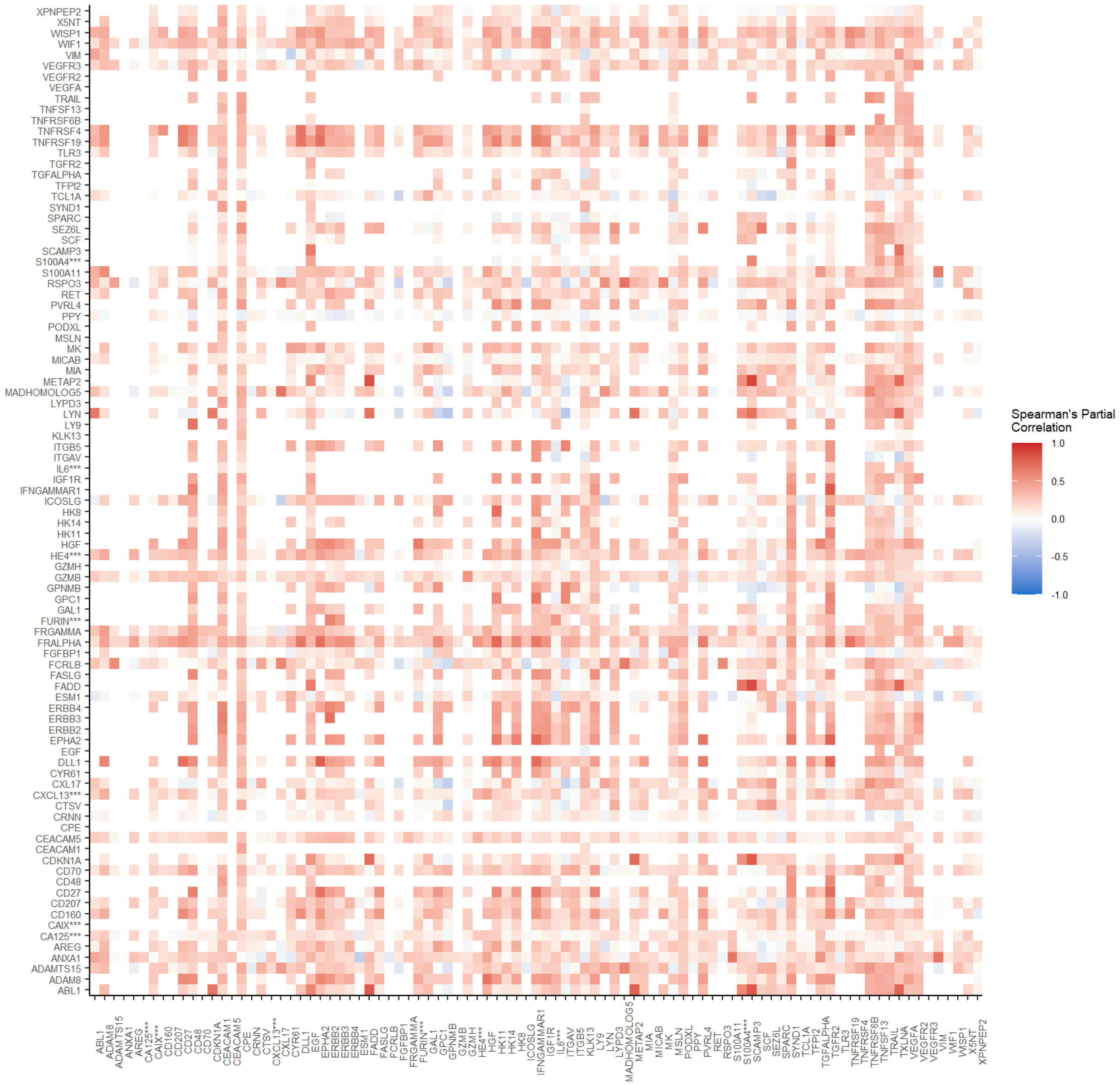

*Controls*

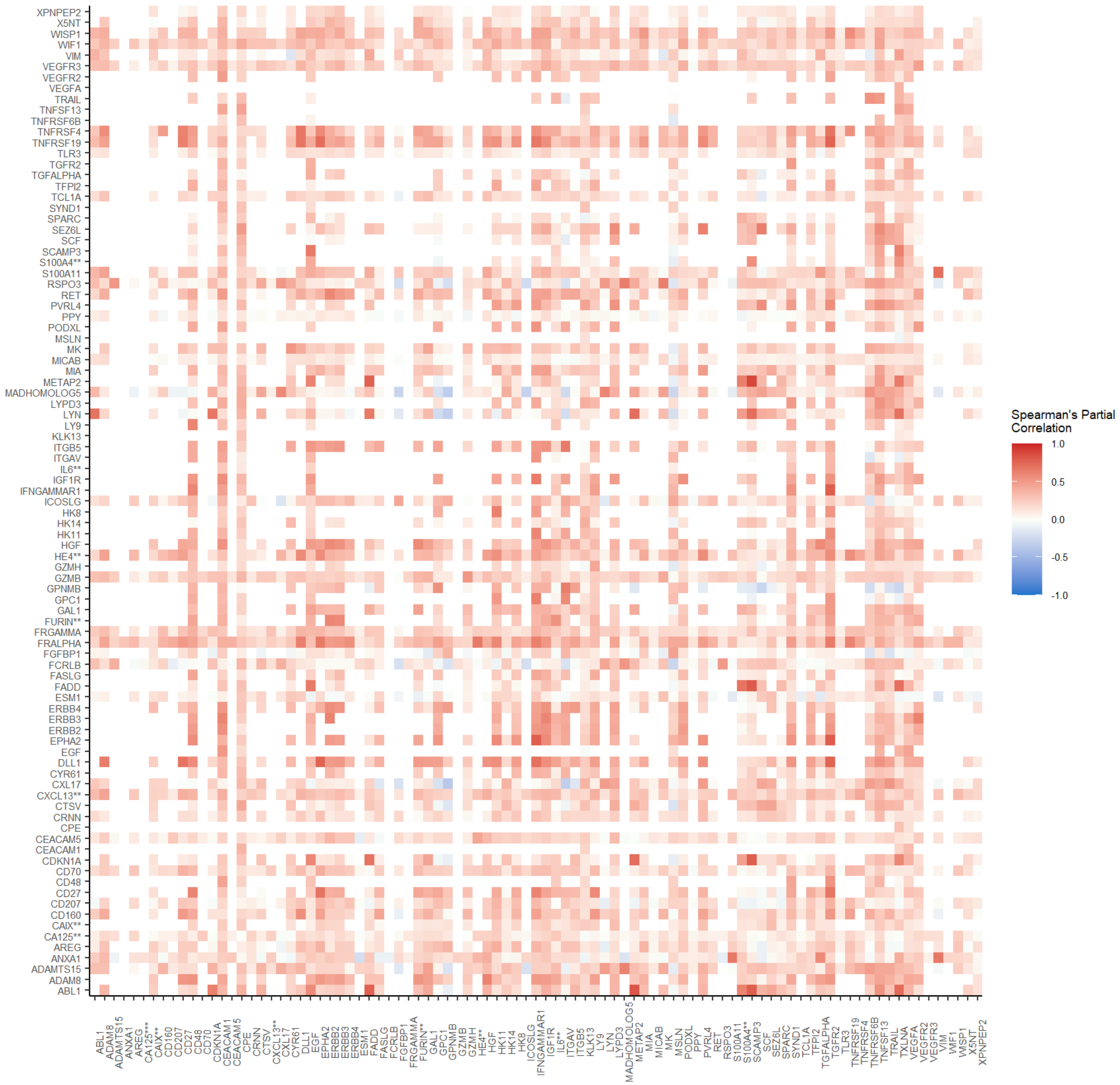


+ Only significant correlations (p<.05) are shown. Proteins selected from the ROC analyses are indicated with "**"

**Supplemental Figure F2.** ROC curves depicting the discriminative performance (AUC (95% CI)) of top performing proteins that did not overlap between the EPIC and Janus cohorts (i.e, top performing proteins found in Janus, but not in EPIC and vice versa). ROC curves for the 12-24 lag-time interval are also displayed. For both the EPIC and Janus cohorts, AUCs are adjusted for age at blood draw, and additionally menopausal status for the EPIC cohort only.

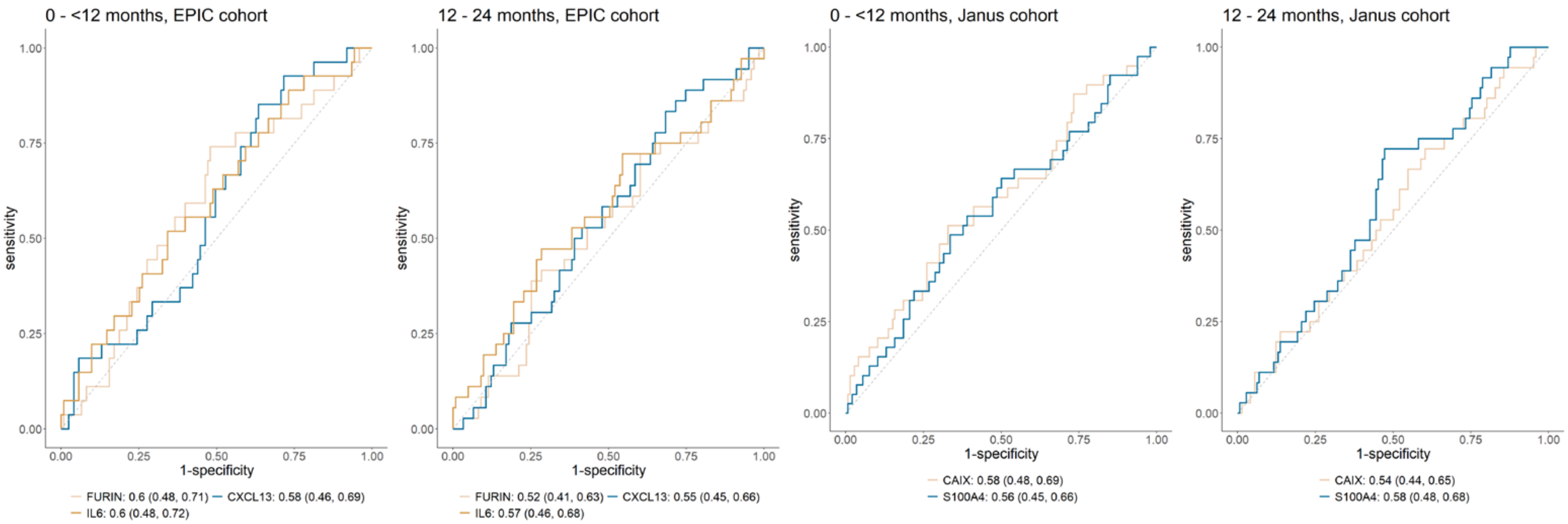

Supplemental Table T1. Discriminative performance (AUC (95% CI)) of all proteins across both the EPIC and Janus cohorts and both lag-times[+]

| Protein | EPIC, 0-<12 mo lag-time | EPIC, 12-24 mo lag-time | Janus, 0-<12 mo lag-time | Janus, 12-24 mo lag-time |
|---|---|---|---|---|
| ABL1 | 0.54 (0.42, 0.65) | 0.53 (0.43, 0.63) | 0.56 (0.46, 0.67) | 0.55 (0.45, 0.65) |
| ADAM8 | 0.61 (0.49, 0.72) | 0.55 (0.45, 0.66) | 0.56 (0.46, 0.67) | 0.62 (0.52, 0.72) |
| ADAMTS15 | 0.55 (0.43, 0.67) | 0.58 (0.48, 0.69) | 0.57 (0.47, 0.67) | 0.5 (0.4, 0.6) |
| ANXA1 | 0.59 (0.46, 0.71) | 0.49 (0.38, 0.6) | 0.57 (0.47, 0.66) | 0.57 (0.47, 0.67) |
| AREG | 0.54 (0.42, 0.65) | 0.54 (0.44, 0.65) | 0.57 (0.47, 0.67) | 0.6 (0.51, 0.7) |
| **CA125** | **0.72 (0.6, 0.83)** | **0.59 (0.48, 0.7)** | **0.74 (0.65, 0.83)** | **0.69 (0.6, 0.77)** |
| **CAIX** | **0.66 (0.53, 0.79)** | **0.58 (0.47, 0.69)** | **0.58 (0.48, 0.69)** | **0.54 (0.44, 0.65)** |
| CD160 | 0.54 (0.42, 0.66) | 0.56 (0.46, 0.67) | 0.56 (0.45, 0.66) | 0.53 (0.43, 0.64) |
| CD207 | 0.52 (0.4, 0.65) | 0.52 (0.41, 0.63) | 0.55 (0.44, 0.66) | 0.53 (0.43, 0.64) |
| CD27 | 0.52 (0.4, 0.64) | 0.58 (0.47, 0.69) | 0.55 (0.45, 0.65) | 0.57 (0.47, 0.68) |
| CD48 | 0.52 (0.4, 0.65) | 0.52 (0.41, 0.63) | 0.55 (0.45, 0.66) | 0.58 (0.48, 0.69) |
| CD70 | 0.5 (0.37, 0.62) | 0.54 (0.43, 0.66) | 0.64 (0.54, 0.74) | 0.56 (0.45, 0.66) |
| CDKN1A | 0.53 (0.41, 0.64) | 0.53 (0.42, 0.63) | 0.55 (0.45, 0.66) | 0.47 (0.38, 0.57) |
| CEACAM1 | 0.6 (0.47, 0.72) | 0.49 (0.38, 0.6) | 0.57 (0.47, 0.67) | 0.6 (0.49, 0.71) |
| CEACAM5 | 0.55 (0.41, 0.68) | 0.56 (0.46, 0.67) | 0.57 (0.48, 0.67) | 0.54 (0.44, 0.64) |
| CPE | 0.56 (0.44, 0.67) | 0.57 (0.47, 0.67) | 0.55 (0.45, 0.66) | 0.54 (0.44, 0.64) |

| Protein | EPIC, 0-<12 mo lag-time | EPIC, 12-24 mo lag-time | Janus, 0-<12 mo lag-time | Janus, 12-24 mo lag-time |
|---|---|---|---|---|
| CRNN | 0.55 (0.42, 0.68) | 0.58 (0.48, 0.68) | 0.59 (0.49, 0.68) | 0.57 (0.47, 0.67) |
| CTSV | 0.52 (0.4, 0.65) | 0.5 (0.39, 0.61) | 0.54 (0.43, 0.65) | 0.54 (0.44, 0.64) |
| **CXCL13** | **0.58 (0.46, 0.69)** | **0.55 (0.45, 0.66)** | **0.67 (0.57, 0.77)** | **0.59 (0.48, 0.7)** |
| CXL17 | 0.59 (0.48, 0.7) | 0.5 (0.39, 0.62) | 0.56 (0.45, 0.67) | 0.57 (0.48, 0.67) |
| CYR61 | 0.54 (0.42, 0.66) | 0.52 (0.41, 0.63) | 0.61 (0.5, 0.71) | 0.57 (0.47, 0.67) |
| DLL1 | 0.59 (0.47, 0.71) | 0.57 (0.45, 0.68) | 0.6 (0.5, 0.7) | 0.57 (0.47, 0.67) |
| EGF | 0.57 (0.45, 0.69) | 0.55 (0.45, 0.65) | 0.55 (0.45, 0.66) | 0.64 (0.54, 0.74) |
| EPHA2 | 0.56 (0.45, 0.68) | 0.64 (0.55, 0.74) | 0.58 (0.48, 0.69) | 0.59 (0.49, 0.69) |
| ERBB2 | 0.62 (0.5, 0.73) | 0.59 (0.47, 0.7) | 0.56 (0.46, 0.66) | 0.56 (0.46, 0.66) |
| ERBB3 | 0.58 (0.46, 0.69) | 0.64 (0.53, 0.75) | 0.57 (0.48, 0.67) | 0.56 (0.45, 0.66) |
| ERBB4 | 0.53 (0.4, 0.66) | 0.57 (0.46, 0.68) | 0.54 (0.44, 0.65) | 0.53 (0.43, 0.63) |
| ESM1 | 0.54 (0.4, 0.69) | 0.55 (0.43, 0.67) | 0.54 (0.43, 0.65) | 0.58 (0.47, 0.68) |
| FADD | 0.55 (0.43, 0.67) | 0.49 (0.37, 0.6) | 0.45 (0.34, 0.55) | 0.55 (0.44, 0.66) |
| FASLG | 0.54 (0.43, 0.66) | 0.53 (0.42, 0.64) | 0.58 (0.48, 0.68) | 0.54 (0.44, 0.64) |
| FCRLB | 0.58 (0.46, 0.7) | 0.56 (0.45, 0.67) | 0.55 (0.45, 0.66) | 0.54 (0.44, 0.64) |
| FGFBP1 | 0.56 (0.45, 0.68) | 0.56 (0.46, 0.67) | 0.63 (0.54, 0.73) | 0.65 (0.55, 0.75) |
| FRALPHA | 0.54 (0.42, 0.66) | 0.64 (0.54, 0.74) | 0.58 (0.47, 0.69) | 0.53 (0.43, 0.63) |

| Protein | EPIC, 0-<12 mo lag-time | EPIC, 12-24 mo lag-time | Janus, 0-<12 mo lag-time | Janus, 12-24 mo lag-time |
|---|---|---|---|---|
| FRGAMMA | 0.51 (0.38, 0.64) | 0.54 (0.43, 0.65) | 0.56 (0.46, 0.66) | 0.56 (0.46, 0.67) |
| **FURIN** | **0.6 (0.48, 0.71)** | **0.52 (0.41, 0.63)** | **0.68 (0.59, 0.77)** | **0.58 (0.48, 0.69)** |
| GAL1 | 0.54 (0.42, 0.66) | 0.57 (0.47, 0.68) | 0.61 (0.51, 0.71) | 0.57 (0.46, 0.68) |
| GPC1 | 0.52 (0.4, 0.65) | 0.57 (0.46, 0.67) | 0.55 (0.45, 0.66) | 0.61 (0.52, 0.71) |
| GPNMB | 0.52 (0.39, 0.64) | 0.46 (0.35, 0.58) | 0.55 (0.44, 0.65) | 0.61 (0.51, 0.71) |
| GZMB | 0.55 (0.42, 0.67) | 0.56 (0.45, 0.67) | 0.54 (0.43, 0.65) | 0.53 (0.43, 0.64) |
| GZMH | 0.57 (0.44, 0.71) | 0.54 (0.43, 0.65) | 0.56 (0.45, 0.67) | 0.55 (0.45, 0.65) |
| **HE4** | **0.76 (0.66, 0.87)** | **0.66 (0.56, 0.77)** | **0.75 (0.65, 0.84)** | **0.62 (0.52, 0.71)** |
| HGF | 0.56 (0.44, 0.68) | 0.48 (0.37, 0.59) | 0.63 (0.52, 0.73) | 0.65 (0.55, 0.74) |
| HK11 | 0.6 (0.48, 0.72) | 0.51 (0.4, 0.62) | 0.62 (0.52, 0.72) | 0.58 (0.47, 0.68) |
| HK14 | 0.55 (0.43, 0.67) | 0.49 (0.38, 0.61) | 0.54 (0.44, 0.65) | 0.56 (0.45, 0.66) |
| HK8 | 0.64 (0.52, 0.75) | 0.53 (0.43, 0.64) | 0.56 (0.45, 0.67) | 0.56 (0.45, 0.67) |
| ICOSLG | 0.53 (0.42, 0.65) | 0.51 (0.39, 0.62) | 0.58 (0.48, 0.68) | 0.66 (0.55, 0.76) |
| IFNGAMMAR1 | 0.52 (0.4, 0.65) | 0.57 (0.46, 0.69) | 0.56 (0.45, 0.66) | 0.64 (0.54, 0.74) |
| IGF1R | 0.55 (0.43, 0.67) | 0.54 (0.43, 0.65) | 0.59 (0.49, 0.7) | 0.63 (0.51, 0.74) |
| **IL6** | **0.6 (0.48, 0.72)** | **0.57 (0.46, 0.68)** | **0.66 (0.57, 0.74)** | **0.59 (0.48, 0.69)** |
| ITGAV | 0.58 (0.46, 0.69) | 0.57 (0.46, 0.68) | 0.61 (0.51, 0.72) | 0.58 (0.48, 0.69) |

| Protein | EPIC, 0-<12 mo lag-time | EPIC, 12-24 mo lag-time | Janus, 0-<12 mo lag-time | Janus, 12-24 mo lag-time |
|---|---|---|---|---|
| ITGB5 | 0.52 (0.4, 0.64) | 0.56 (0.46, 0.66) | 0.55 (0.45, 0.66) | 0.59 (0.49, 0.7) |
| KLK13 | 0.57 (0.44, 0.69) | 0.54 (0.44, 0.64) | 0.58 (0.47, 0.68) | 0.53 (0.43, 0.63) |
| LY9 | 0.55 (0.42, 0.67) | 0.53 (0.43, 0.64) | 0.56 (0.46, 0.67) | 0.58 (0.48, 0.68) |
| LYN | 0.51 (0.39, 0.63) | 0.48 (0.38, 0.59) | 0.57 (0.47, 0.68) | 0.54 (0.44, 0.64) |
| LYPD3 | 0.59 (0.47, 0.71) | 0.62 (0.53, 0.72) | 0.55 (0.44, 0.65) | 0.55 (0.45, 0.65) |
| MADHOMOLOG5 | 0.55 (0.43, 0.67) | 0.51 (0.4, 0.62) | 0.54 (0.44, 0.65) | 0.54 (0.44, 0.65) |
| METAP2 | 0.53 (0.41, 0.65) | 0.5 (0.39, 0.61) | 0.58 (0.47, 0.7) | 0.43 (0.33, 0.52) |
| MIA | 0.53 (0.39, 0.66) | 0.58 (0.47, 0.7) | 0.61 (0.51, 0.71) | 0.55 (0.44, 0.66) |
| MICAB | 0.52 (0.39, 0.65) | 0.49 (0.38, 0.6) | 0.55 (0.45, 0.66) | 0.56 (0.46, 0.66) |
| MK | 0.54 (0.41, 0.68) | 0.47 (0.35, 0.58) | 0.63 (0.53, 0.72) | 0.57 (0.47, 0.67) |
| MSLN | 0.53 (0.42, 0.65) | 0.52 (0.42, 0.63) | 0.56 (0.45, 0.66) | 0.6 (0.5, 0.7) |
| PODXL | 0.57 (0.43, 0.71) | 0.57 (0.46, 0.68) | 0.46 (0.35, 0.56) | 0.57 (0.47, 0.67) |
| PPY | 0.59 (0.47, 0.72) | 0.54 (0.43, 0.64) | 0.59 (0.49, 0.7) | 0.53 (0.43, 0.63) |
| PVRL4 | 0.47 (0.34, 0.6) | 0.43 (0.32, 0.55) | 0.55 (0.44, 0.66) | 0.57 (0.47, 0.67) |
| RET | 0.6 (0.47, 0.72) | 0.55 (0.43, 0.66) | 0.56 (0.45, 0.66) | 0.53 (0.43, 0.63) |
| RSPO3 | 0.6 (0.48, 0.73) | 0.55 (0.44, 0.66) | 0.59 (0.49, 0.69) | 0.54 (0.44, 0.64) |
| S100A11 | 0.54 (0.42, 0.66) | 0.54 (0.44, 0.65) | 0.56 (0.46, 0.66) | 0.56 (0.46, 0.66) |

| Protein | EPIC, 0-<12 mo lag-time | EPIC, 12-24 mo lag-time | Janus, 0-<12 mo lag-time | Janus, 12-24 mo lag-time |
|---|---|---|---|---|
| **S100A4** | **0.65 (0.54, 0.77)** | **0.49 (0.38, 0.6)** | **0.56 (0.45, 0.66)** | **0.58 (0.48, 0.68)** |
| SCAMP3 | 0.56 (0.44, 0.69) | 0.53 (0.42, 0.63) | 0.57 (0.47, 0.68) | 0.57 (0.47, 0.67) |
| SCF | 0.48 (0.36, 0.61) | 0.45 (0.33, 0.56) | 0.6 (0.5, 0.69) | 0.54 (0.44, 0.64) |
| SEZ6L | 0.57 (0.44, 0.7) | 0.6 (0.48, 0.72) | 0.57 (0.47, 0.67) | 0.54 (0.43, 0.64) |
| SPARC | 0.57 (0.44, 0.69) | 0.54 (0.43, 0.66) | 0.55 (0.45, 0.65) | 0.55 (0.44, 0.65) |
| SYND1 | 0.56 (0.42, 0.7) | 0.54 (0.43, 0.66) | 0.58 (0.47, 0.69) | 0.61 (0.5, 0.71) |
| TCL1A | 0.52 (0.39, 0.64) | 0.56 (0.46, 0.66) | 0.56 (0.45, 0.66) | 0.59 (0.49, 0.69) |
| TFPI2 | 0.59 (0.46, 0.72) | 0.61 (0.51, 0.72) | 0.58 (0.47, 0.69) | 0.59 (0.49, 0.68) |
| TGFALPHA | 0.57 (0.45, 0.7) | 0.54 (0.43, 0.64) | 0.6 (0.49, 0.7) | 0.6 (0.5, 0.69) |
| TGFR2 | 0.57 (0.45, 0.69) | 0.58 (0.47, 0.69) | 0.59 (0.49, 0.69) | 0.61 (0.51, 0.72) |
| TLR3 | 0.62 (0.5, 0.74) | 0.53 (0.42, 0.65) | 0.55 (0.44, 0.66) | 0.54 (0.44, 0.64) |
| TNFRSF19 | 0.57 (0.45, 0.69) | 0.58 (0.47, 0.69) | 0.58 (0.47, 0.68) | 0.57 (0.46, 0.67) |
| TNFRSF4 | 0.58 (0.46, 0.7) | 0.6 (0.5, 0.71) | 0.58 (0.48, 0.69) | 0.54 (0.44, 0.64) |
| TNFRSF6B | 0.56 (0.44, 0.68) | 0.63 (0.52, 0.73) | 0.55 (0.43, 0.66) | 0.56 (0.46, 0.66) |
| TNFSF13 | 0.53 (0.4, 0.66) | 0.6 (0.5, 0.71) | 0.6 (0.5, 0.7) | 0.55 (0.44, 0.65) |
| TRAIL | 0.54 (0.42, 0.67) | 0.54 (0.43, 0.64) | 0.59 (0.48, 0.69) | 0.55 (0.45, 0.66) |
| TXLNA | 0.6 (0.48, 0.72) | 0.49 (0.38, 0.6) | 0.55 (0.45, 0.66) | 0.46 (0.36, 0.56) |

| Protein | EPIC, 0-<12 mo lag-time | EPIC, 12-24 mo lag-time | Janus, 0-<12 mo lag-time | Janus, 12-24 mo lag-time |
|---|---|---|---|---|
| VEGFA | 0.56 (0.44, 0.68) | 0.58 (0.47, 0.68) | 0.55 (0.44, 0.66) | 0.59 (0.49, 0.69) |
| VEGFR2 | 0.52 (0.39, 0.65) | 0.59 (0.5, 0.69) | 0.55 (0.46, 0.65) | 0.56 (0.45, 0.66) |
| VEGFR3 | 0.53 (0.41, 0.64) | 0.55 (0.44, 0.65) | 0.46 (0.36, 0.56) | 0.6 (0.49, 0.7) |
| VIM | 0.57 (0.45, 0.69) | 0.53 (0.43, 0.63) | 0.56 (0.46, 0.66) | 0.61 (0.52, 0.71) |
| WIF1 | 0.49 (0.35, 0.63) | 0.54 (0.43, 0.65) | 0.62 (0.52, 0.73) | 0.6 (0.5, 0.7) |
| WISP1 | 0.54 (0.41, 0.66) | 0.61 (0.5, 0.71) | 0.54 (0.44, 0.65) | 0.59 (0.49, 0.69) |
| X5NT | 0.64 (0.52, 0.76) | 0.54 (0.43, 0.64) | 0.61 (0.52, 0.71) | 0.53 (0.43, 0.63) |
| XPNPEP2 | 0.49 (0.36, 0.62) | 0.53 (0.42, 0.64) | 0.58 (0.47, 0.68) | 0.53 (0.43, 0.63) |

[+]Proteins with an AUC of >= 0.65 in the 0-<12 lag-time window and selected for final analyses are bolded

**Supplemental Figure F3.** Top performing protein levels by time before endometrial cancer diagnosis. Locally Estimated Scatterplot Smoothing (LOESS) used to produce fitted lines.

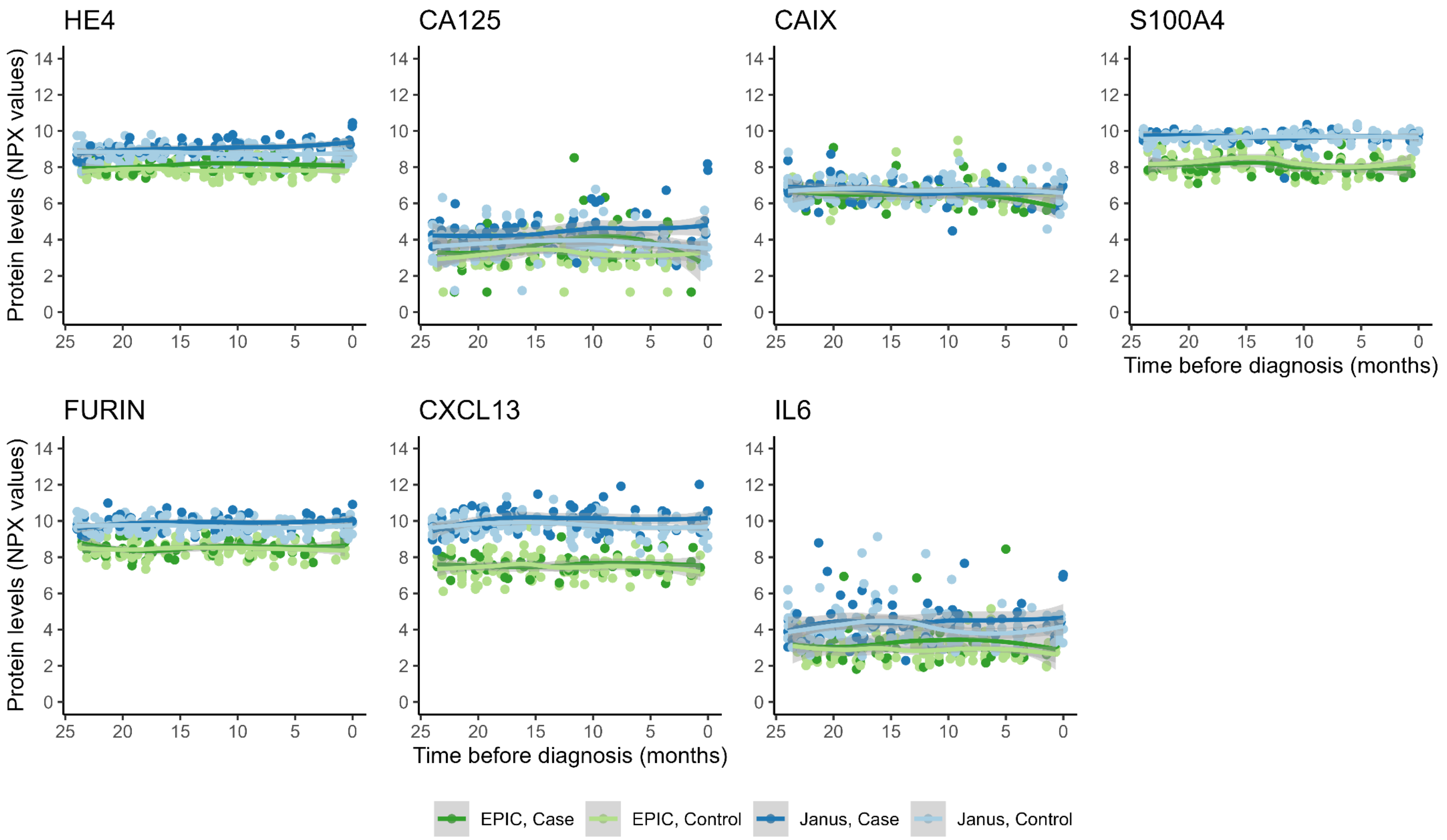

Supplement: Supplementary file 1 — Data S1. Supporting Information. [file IJC-157-480-s001.pdf]
